# Effects of a parent advocacy intervention on service access for transition‐aged autistic youth: a multisite randomized controlled trial

**DOI:** 10.1111/jcpp.70036

**Published:** 2025-08-17

**Authors:** Julie Lounds Taylor, Leann Smith DaWalt, Meghan M. Burke, Meng Xu, James C. Slaughter

**Affiliations:** ^1^ Vanderbilt University Medical Center Nashville TN USA; ^2^ University of Wisconsin‐Madison Madison WI USA; ^3^ Vanderbilt University Nashville TN USA

**Keywords:** Autism, services, transition to adulthood, randomized controlled trial

## Abstract

**Background:**

Autistic youth in the United States face many challenges accessing services as they transition to adulthood. Improving parents' ability to advocate for services is a promising way to improve service access. The current study tested whether participation in an intervention to improve parents' ability to advocate for adult services (called Advocating for Supports to Improve Service Transitions or ASSIST) led to increased service access for their transition‐aged autistic youth.

**Methods:**

Using a multisite, single‐blind parallel‐group design, we randomized 185 parents of transition‐aged autistic youth to either a treatment condition that received the ASSIST intervention, or a control condition that received comprehensive written information about adult services. Primary outcomes for this report – number of government programs that fund services and direct services received by the youth – were collected via parental interview at baseline, six, and 12 months after intervention.

**Results:**

Primary analyses found no significant treatment effects on service access. Subgroup analyses, however, detected treatment effects for families of youth who had exited high school prior to their families taking ASSIST. Among those families, youth from the treatment group were receiving more government programs that fund services at 6 months after intervention compared with youth from the control group.

**Conclusions:**

We cannot conclude from our findings that ASSIST improved access to services, though there was some evidence to suggest increased access to government programs that fund services for families of autistic youth who had exited high school. Future research should investigate which families can translate written information about adult services (i.e. the control condition) into improved service access, and which families need more individualized support beyond a group‐based class to see improvements in service access.

## Introduction

The transition from high school into adulthood is a challenging time for autistic youth, with many experiencing difficulties in employment, postsecondary education, independent living, mental health, and social relationships (Hollocks, Lerh, Magiati, Meiser‐Stedman, & Brugha, 2019; Orsmond, Shattuck, Cooper, Sterzing, & Anderson, 2013; Shattuck et al., 2012; Taylor & Seltzer, 2012). Access to adult disability services can help mitigate these challenges (Alverson & Yamamoto, 2016; Roux, Rast, Anderson, Garfield, & Shattuck, 2021; Taylor & Mailick, 2014); however, in the United States, services become more difficult to access after autistic youth leave high school (Laxman, Taylor, DaWalt, Greenberg, & Mailick, 2019; Shattuck, Wagner, Narendorf, Sterzing, & Hensley, 2011). After high school exit, youth encounter an adult disability service system that is inadequately funded, fragmented, and difficult to navigate. Understanding each agency's eligibility scheme, funding mechanism, and programming can be a significant barrier to procuring needed services and supports.

Teaching families how to advocate for adult disability services is a promising way to improve service access for autistic youth. Many studies have acknowledged that parental advocacy is a key factor in service access for school‐aged children with disabilities (e.g. Szlamka, Tekola, Hoekstra, & Hanlon, 2022; Taylor, Wright, Pothier, Hill, & Roserberg, 2019; Trainor, 2008; Turnbull & Turnbull, 2001), and our research suggests that it remains important during the transition to adulthood. Specifically, we found that parental advocacy activities were one of the strongest predictors of the number of services that autistic transition‐aged youth received (Lee, Burke, DaWalt, Li, & Taylor, 2022). Yet parents of autistic youth are often uninformed about the adult service system (Gilson, Bethune, Carter, & McMillan, 2017), hampering their ability to advocate effectively for services to improve transition outcomes for their youth.

To address this need, we developed an intervention to support families of autistic youth in accessing services during the transition to adulthood by enhancing parents' ability to advocate for adult services and supports. The intervention – called Advocating for Supports to Improve Service Transitions or ASSIST – was delivered in three states in the United States and tested using a randomized controlled trial (RCT) design. To date, we have studied implementation outcomes and intervention effects on the treatment target of parent advocacy ability. We found that ASSIST was delivered across sites with high feasibility and treatment fidelity and was acceptable to families (Taylor et al., 2022). Consistent with advocacy interventions focused on school‐based services and supports (e.g. Burke, Goldman, & Li, 2023; Goldman, Goscicki, Burke, & Hodapp, 2020), relative to the control group, families who participated in ASSIST had greater gains in advocacy ability (i.e. the treatment target; Taylor, DaWalt, Burke, Slaughter, & Xu, 2023). The current study represents the next step in this line of research, by testing whether participating in ASSIST improved service access (a prespecified primary outcome of the RCT) during the transition to adulthood for autistic youth. Though we would expect that participating in an intervention to increase parents' ability to advocate for services would lead to better service access for their youth, there have been no studies to our knowledge that have tested this directly.

### The present study

The present study investigated whether, relative to the control group who received access to comprehensive written information, families who participated in the ASSIST intervention demonstrated greater access to government programs and the services funded by those programs at six months and 12 months after receiving the intervention. We examined both government programs and direct services as they represent distinct dimensions of access. Receipt of government programs is often necessary to fund direct services, but they are not analogous to the receipt of such services; for example, families may not be able to access the services that are the target of funding (e.g. funding for a personal support worker but no personnel available in their area to do the work). We hypothesized that families who participated in ASSIST would experience greater gains in services relative to control group families.

In addition to our primary analyses of treatment group differences, we conducted prespecified secondary analyses in which we examined potential treatment effects in subgroups. Specifically, we considered whether the youth was in high school versus had exited high school and whether the autistic youth had a co‐occurring intellectual disability. These factors are critical to fully consider for service access. Some of the government programs discussed in ASSIST are relevant for youth once they turn 18 years of age or have left the school system. Thus, the potential to translate information gained from ASSIST into service gain is qualitatively different for those in high school (who can use the information to plan for the future) relative to those out of high school (who can use the information immediately). Similarly, autistic youth without intellectual disability typically have access to far fewer services than autistic youth with co‐occurring intellectual disability (Lai & Weiss, 2017; Shattuck et al., 2011; Taylor & Henninger, 2015); the information about service access may be less immediately actionable for those without intellectual disability. Given this information, we hypothesized that families of youth with intellectual disability and families of youth who had exited high school would see gains in service access after taking the ASSIST intervention relative to the control group.

## Method

### Participants

The following eligibility criteria were used for the RCT: (a) Participant was a parent or legal guardian of an autistic youth who was between the ages of 16 and 26 years (inclusive); (b) parent provided documentation of the youth's autism diagnosis; (c) parent was able to attend the ASSIST sessions and lived in a state where one of the study sites was located (Illinois, Tennessee, Wisconsin); (d) youth had lifetime scores on the Social Communication Questionnaire (Rutter, Bailey, & Lord, 2003; a parent‐report screener for autism) that suggested they were likely to meet clinical criteria for autism.^1^ In terms of youth age, we set a lower bound of age 16 to reflect when transition planning is mandated to have begun in the schools (idea.ed.gov), and an upper bound of age 26 to reflect youth who are still in the “transition years” as defined by the Institute of Medicine (IOM & NRC, 2014). Families were recruited through a number of venues including research registries and other studies, disability agencies, schools, and autism organizations/groups.

**Figure 1:**
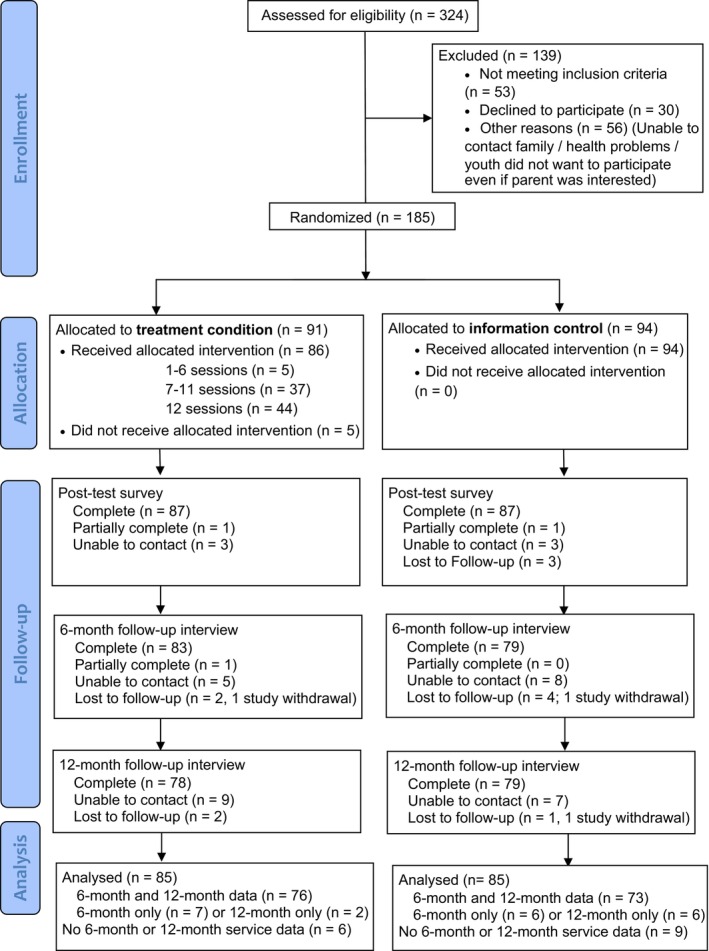
CONSORT flow diagram

The CONSORT flow diagram is shown in Figure 1. One hundred eighty‐five participants were recruited across three study sites (Illinois = 61, Tennessee = 63, Wisconsin = 61); 91 were assigned to the treatment condition and 94 to the control condition. Of the 185, 163 (88.1%) were retained at the six‐month follow‐up and 157 (84.9%) were retained at the 12‐month follow‐up. Eight participants did not participate in the six‐month follow‐up but did participate in the 12‐month follow‐up. Participants were included in this analysis if they had data collected at either of these time points (see Figure 1); the sample size of 170 included 85 in the treatment condition (93.4% retention) and 85 in the control condition (90.4% retention).

Baseline demographic characteristics for participants included in this analysis by experimental condition are presented in Table 1. Youth in both conditions averaged 19.5 years of age; about three‐quarters were male. About 60% were in high school at baseline and just under 40% had an intellectual disability. About 90% of parent participants were female. There was limited diversity in youth race or parent education. There were no statistically significant differences between treatment and control participants in demographic variables or the number of government programs and direct services that youth were receiving at baseline (as reported in Taylor et al., 2023, there were also no significant group differences in these variables among the full randomized sample).

**Table 1:** Baseline characteristics by experimental condition for the analytic sample

	Treatment	Control
	*N* = 85	*N* = 85
	% (*n*) or *M* (*SD*)	% (*n*) or *M* (*SD*)
Youth characteristics		
Age in years	19.57 (2.88)	19.52 (2.61)
Gender		
Male	78% (66)	73% (62)
Female	22% (19)	25% (21)
Nonbinary gender or other	0%	1% (1)
I am not sure/I prefer not to respond	0%	1% (1)
Race		
White	68% (58)	78% (66)
Black/African American	8% (7)	8% (7)
Asian	8% (7)	4% (3)
More than one race	13% (11)	7% (6)
Other	2% (2)	4% (3)
Hispanic/Latino ethnicity	9% (8)	11% (9)
In high school	59% (50)	60% (51)
Has intellectual disability	39% (33)	38% (32)
Cohort		
Cohort one	48% (41)	45% (38)
Cohort two	52% (44)	55% (47)
Site		
Tennessee	37% (31)	33% (28)
Illinois	32% (27)	33% (28)
Wisconsin	32% (27)	34% (29)
Parent characteristics		
Age in years	52.10 (6.75)	50.27 (5.87)
Gender		
Male	12% (10)	8% (7)
Female	88% (75)	92% (78)
Highest level of education		
Less than high school	0%	0%
High school degree (or equivalent)	5% (4)	5% (4)
Some college	18% (15)	14% (12)
Associates degree	13% (11)	8% (7)
Bachelor's degree	36% (31)	38% (32)
Master's degree	27% (23)	27% (23)
Ph.D. or Professional degree (JD, MD, etc.)	4% (4)	8% (7)

### Procedures

The trial is registered in ClinicalTrials.gov, # NCT04173663. This was a multisite, single‐blind parallel‐group study conducted in the United States across three sites. Participants were recruited from December 2019 to November 2020. After recruitment, screening, and informed consent, baseline data were collected from parents and autistic youth via interview and questionnaire. Interviews were conducted either in person at the respective universities/clinics or via web conferencing. After completing baseline data collection, participants were randomized to the treatment or control condition (1:1 ratio) using a computer‐generated list of random numbers created by a biostatistician (who was not involved in data collection or allocation to experimental condition). To encourage equivalent distribution of factors known to impact service access, block randomization was used within each site stratified by whether the autistic youth was in high school and had intellectual disability. These blocking variables are also used to stratify the sample into subgroups for the secondary analysis. Study personnel collecting baseline data were blinded to future group assignments.

Participants were recruited and interventions were delivered in two cohorts at each site, for a total of six groups. Treatment for cohort one started in February 2020 (Tennessee, Illinois) or August 2020 (Wisconsin). Treatment for cohort two started in August 2020 (Tennessee, Illinois) or November 2020 (Wisconsin). Though multiple family members were allowed to participate in the intervention, one was designated as the primary study respondent and asked to attend all sessions and complete data collection. Six months after treatment groups finished the ASSIST intervention, parents completed a telephone or video‐conferencing interview that included information on the government programs and direct services their youth was receiving. The data collection window for the six‐month follow‐up opened 5 months after completing ASSIST and closed at the end of the seventh month (resulting in a three‐month window). This parent interview was repeated at 12 months after finishing the ASSIST intervention (termed the 12‐month follow‐up). Though information from the parent interview is the focus of this report, the 12‐month data collection also included questionnaires completed by parents and by the autistic youth, and a brief interview with the youth. The data collection window for the 12‐month follow‐up opened 11 months after completing ASSIST and closed at the end of the 13^th^ month (resulting in a three‐month window). Though participants were not blinded, all research staff administering the follow‐up interviews and facilitating completion of the surveys were blinded to experimental condition. Adverse events were monitored and reported through the study.

### Experimental conditions

Full information on experimental conditions and the impact of COVID‐19 on intervention delivery can be found in Taylor et al. (2022, 2023). The *treatment condition* participated in the ASSIST program, which was primarily delivered virtually due to the COVID‐19 pandemic. As part of the ASSIST program, materials that would be covered in each session (i.e. handouts of the slides from the presentations, resources, tipsheets) were mailed to all treatment group participants a week prior to that session. The *control condition* received the same written materials as the treatment condition on the same schedule and thus had access to the same information as the treatment group. The treatment group, however, also had the opportunity to hear an explanation of the materials, ask questions to the presenters (who had expertise on the topic at hand; see below), and participate in group‐based discussions with the presenters and other group members. Control condition participants were able to take the ASSIST program after a 12‐month waiting period.

### Intervention: ASSIST

Full information on the development, content, and implementation of ASSIST can be found in Taylor et al. (2022). ASSIST is a 12‐week group‐based program that teaches parents about the adult disability service system in their state, including the range of services that may be available to them as their autistic youth transitions to adulthood, how these services might benefit their youth, and the most effective ways to pursue services and supports. ASSIST was led by a facilitator from the community (i.e. a member of a local disability organization), with support provided by the university team as needed. Each weekly session lasted 2 hr (24 hr total) and included topics related to person‐centered planning and thinking, legal protections, income supports, health insurance, employment, postsecondary education, housing, supports for community participation, and advocacy. ASSIST focuses not only on providing information about various adult services and supports, but also on helping participants understand how these complex service systems can support their youth.

A description of the structure of ASSIST sessions can be found in Taylor et al. (2022, 2023). ASSIST sessions feature both didactic instruction through videos and live presentations, as well as opportunities to synthesize the information via group discussions and opportunities to ask questions. An important feature of ASSIST is its inclusion of standardized, national information that is consistent across all implementations, balanced with site‐specific information that is relevant to the state in which the program is delivered. Because the adult service system is different in each state in the United States, it would not be effective to only present standardized, nationally relevant information; doing so would exclude state‐specific programs and regulations, severely limiting the information that could be provided to participants. Alternatively, some standardized curriculum is necessary to ensure that participants are getting comparable information regardless of where the intervention is delivered. To strike this balance, nationally relevant information is presented at the start of each session via a brief video that covers introductory concepts and terminology related to the topic at hand. Learning objectives are then used to guide the presentation of information that requires state specificity. For each session, a local guest speaker with expertise on that topic delivers a presentation on the state‐specific information, following the learning objectives which remain constant across all sites. For example, Medicaid Waiver programs, which provide long‐term services for people with disabilities to live in their communities, are vastly different across states. The video for this session presents nationally relevant information such as the history of Medicaid long‐term services and supports, and what a person is “waiving” when they get Medicaid waiver services. The state‐specific learning objectives cover the Medicaid Waiver programs available in the state in which ASSIST is being delivered. Learning objectives for each ASSIST session can be found in Taylor et al. (2022).

### Measures

#### Services

Service access – the focus of this report – was prespecified as a primary outcome measure (NCT04173663). We examined services in two ways: the number of government programs that families were receiving and the number of direct services that families were receiving. The same services measures were administered to parents at the baseline, six‐month, and 12‐month follow‐up interviews.

The measure of government programs was developed for this study by generating a list of all state and federal programs that fund adult disability or related services that were covered as part of the ASSIST curriculum. Ten government programs were queried: Supplemental Security Income, Social Security Disability Insurance, Vocational Rehabilitation services, Medicaid waiver services, Medicaid long‐term services and supports, legal protections (e.g. conservatorship, power of attorney), special needs trusts and ABLE accounts, housing choice vouchers (“Section 8”), Supplemental Nutrition Assistance Program, and Medicaid or Medicare health insurance. For each government program, parents were asked if their youth was receiving the program, and this information was used to generate a count of the *number of government programs received* at each time point. When querying about government programs, interviewers had a standardized set of prompts for each program that provided more information for families who were unsure if they were receiving a government program (i.e. they did not know what the program was called). Even with these prompts, interviewers noted that families had a difficult time distinguishing between Medicaid waivers and Medicaid long‐term services and supports. Thus, we combined those two government programs into one item, resulting in a possible range from 0 to 9.

The *number of direct services* the autistic youth was receiving was collected from parents at each time point using questions from the National Longitudinal Transition Study‐2 (Newman et al., 2011). Participants responded either 1 = *yes* or 0 = *no* to whether they were currently receiving each of 21 services (e.g. speech/language services, psychological/mental health services or counseling, transportation; see Lee et al., 2022 for a full list of services). From this information, we generated a count of the number of direct services that the youth was receiving at each time point, with a possible range from 0 to 21.

It is important to note that some specific services are more or less relevant to those with different characteristics; for example, some services are only available to youth who are over 18 or who have left high school, and others are much more difficult to get once youth are out of high school. By including a range of different services in our service count variables, we ensure that services relevant to each subgroup of autistic youth are captured.

*Demographic and behavioral measures* were collected at baseline from parents and autistic youth to characterize the sample. Youth information included gender, race, ethnicity, and whether they had exited the school system. Co‐occurring intellectual disability was determined by consensus using all available information at baseline, including the adaptive behavior composite score from the Vineland Scales of Adaptive Behavior‐III (Sparrow, Saulnier, Cicchetti, & Doll, 2016), information and documentation provided by parents (e.g. Individualized Education Program, clinical records), and IQ scores from the Weschler Abbreviated Scales of Intelligence (Wechsler, 1999; available for 57 youth whose baseline data were collected in‐person prior to COVID‐19). Parent information included gender and education (1 = less than a high school degree to 7 = Ph.D. or professional degree).

### Analysis plan

Our targeted sample size for recruitment (*n* = 180) was determined based on a power calculation using treatment effects observed in our pilot research (Taylor, Hodapp, Burke, Waitz‐Kudla, & Rabideau, 2017) and an expected 85% retention rate through the 12‐month follow‐up (which we met); the sample size was sufficient to detect a 0.17 standardized difference between treatment groups (80% power, two‐sided significance level of 0.05).

Initial descriptive analysis of baseline demographic variables was done by experimental condition to identify if important imbalances in covariates existed among those eligible for this analysis. For our primary analyses, we prespecified a common regression framework to estimate and test for a significant effect of treatment on outcomes at follow‐up. Separate proportional odds ordinal logistic regression models were fit for each outcome at the six‐month or 12‐month follow‐up using an indicator variable for experimental condition (intent to treat; predictor of interest) while controlling for site (Illinois, Tennessee, Wisconsin; 2 *df*), cohort (cohort one vs. cohort two), and the baseline measure of the outcome to improve precision of the estimated treatment effect. Results for treatment effects are presented as fold change in the odds of having more services between experimental conditions with corresponding 95% confidence intervals. Analyses were conducted using the R statistical package.

Given our interest in understanding the service access experiences of subgroups (see introduction), secondary analyses were run in which we repeated the regression models above but stratified the sample into subgroups based on whether the youth was in versus out of high school, and whether the youth did or did not have an intellectual disability. To aid interpretation, we present scatterplots of baseline versus follow‐up scores on each outcome, with fitted regression lines by treatment group for simpler models that do not control for site or cohort.

Note that, although we had preregistered moderational analyses to examine treatment effects in subgroups, it became clear during data collection that service access was qualitatively different for youth in high school versus out of high school and those with versus without intellectual disability. Thus, instead of moderational analyses, we stratified the sample into subgroups based on these two key factors and examined treatment effects within the subgroups.

## Results

### Primary analyses: treatment effects

Table 2 presents estimates from the models investigating treatment effects on services at six‐month and 12‐month follow‐ups, controlling for baseline number of services, study site, and study cohort. Treatment group effects on government programs and direct services at both follow‐up data collections (six‐month, 12‐month) were not statistically significant. We observed a significant site effect on direct services at the 12‐month follow‐up; controlling for baseline services, participants in Wisconsin had a 2.71‐fold increased odds of receiving more services at follow‐up relative to participants in Tennessee.

**Table 2:** Models for primary analyses examining treatment effects on service outcomes

	Six‐month follow‐up			12‐month follow‐up		
	Odds ratio	95% CI Odds ratio	Chi‐square (*df*)	Odds ratio	95% CI Odds ratio	Chi‐square (*df*)
(a) Government programs						
Baseline government programs	5.74	4.24–7.76	128.36 (1)***	4.78	3.61–6.33	119.54 (1)***
Treatment vs. Control^r^ group	1.11	0.62–1.97	0.12 (1)	1.24	0.69–2.22	0.50 (1)
Site			1.89 (2)			1.11 (2)
Illinois vs. Tennessee^r^	1.58	0.77–3.25		1.45	0.71–2.97	
Wisconsin vs. Tennessee^r^	1.03	0.50–2.13		1.33	0.63–2.83	
Cohort one vs. Cohort two^r^	1.59	0.89–2.84	2.46 (1)	1.37	0.76–2.46	1.08 (1)
(b) Direct services						
Baseline direct services	3.39	2.66–4.32	96.90 (1)***	3.15	2.47–4.01	86.93 (1)***
Treatment vs. Control^r^ group	1.46	0.84–2.54	1.81 (1)	0.85	0.49–1.48	0.34 (1)
Site			4.18 (2)			7.35 (2)*
Illinois vs. Tennessee^r^	0.87	0.42–1.82		1.43	0.68–2.99	
Wisconsin vs. Tennessee^r^	1.71	0.86–3.41		2.71	1.29–5.68	
Cohort one vs. Cohort two^r^	0.70	0.40–1.23	1.52 (1)	0.59	0.33–1.03	3.42 (1)

CI, confidence interval; ^r^, reference group; *SE*, standard error.

*p < .05; **p < .01; ***p < .001.

Though primary analyses did not detect treatment group differences in services at the six‐month or 12‐month follow‐ups, supporting analyses (see Table S1) found that both treatment and control groups had statistically significant gains in government programs from baseline to each of the follow‐up data collections.

### Secondary analyses: treatment effects within subgroups

#### Government programs

Table 3 presents estimates from the four subgroup regression models (i.e. those in high school at baseline, those out of high school at baseline, those with intellectual disability, those without intellectual disability) predicting government programs at the six‐month follow‐up. As can be seen from the model, there was a statistically significant treatment effect on the number of government programs at the six‐month follow‐up, but only for families of youth out of high school at baseline. After accounting for baseline government programs, study site, and cohort, youth who were out of high school whose families were in the treatment condition had a 3.81‐fold increased odds (95% CI: 1.34–10.82) of receiving more services than youth out of high school whose families were in the control condition (frequencies of gains in individual government programs are presented in Supporting Information). The treatment effect on six‐month government programs was not statistically significant for youth in high school at baseline, and we did not observe a statistically significant treatment effect when stratifying the sample by whether the autistic youth had an intellectual disability.

**Table 3:** Models examining treatment effects on government programs at six‐month follow‐up, stratified by high school exit and intellectual disability status

(a) Stratified by high school exit						
	In high school			Out of high school		
	Odds ratio	95% CI odds ratio	Chi‐square (*df*)	Odds ratio	95% CI odds ratio	Chi‐square (*df*)
Baseline government programs	5.55	3.71–8.29	70.03 (1)***	9.53	5.19–17.52	52.68 (1)***
Treatment vs. Control^r^ group	0.64	0.30–1.36	1.33 (1)	3.81	1.34–10.82	6.29 (1)*
Site			2.12 (2)			0.07
Illinois vs. Tennessee^r^	1.86	0.75–4.64		1.06	0.30–3.76	
Wisconsin vs. Tennessee^r^	1.02	0.37–2.83		0.90	0.26–3.11	
Cohort one vs. Cohort two^r^	1.07	0.49–2.36	0.03 (1)	3.70	1.29–10.59	5.94*

(b) Stratified by intellectual disability						
	Has intellectual disability			No intellectual disability		
	Odds ratio	95% CI odds ratio	Chi‐square (*df*)	Odds ratio	95% CI odds ratio	Chi‐square (*df*)
Baseline government programs	7.05	4.09–12.17	49.33 (1)***	5.48	3.70–8.11	72.20 (1)***
Treatment vs. Control^r^ group	1.33	0.51–3.51	0.34 (1)	1.00	0.48–2.09	0.00 (1)
Site			4.60 (2)			0.19 (2)
Illinois vs. Tennessee^r^	3.72	0.90–15.43		1.21	0.49–2.97	
Wisconsin vs. Tennessee^r^	0.90	0.27–2.98		1.18	0.45–3.11	
Cohort one vs. Cohort two^r^	1.88	0.69–5.11	1.51 (1)	1.43	0.68–3.01	0.88 (1)

CI, confidence interval; ^r^, reference group.

*p < .05; **p < .01; ***, *p* < .001.

**Figure 2:**
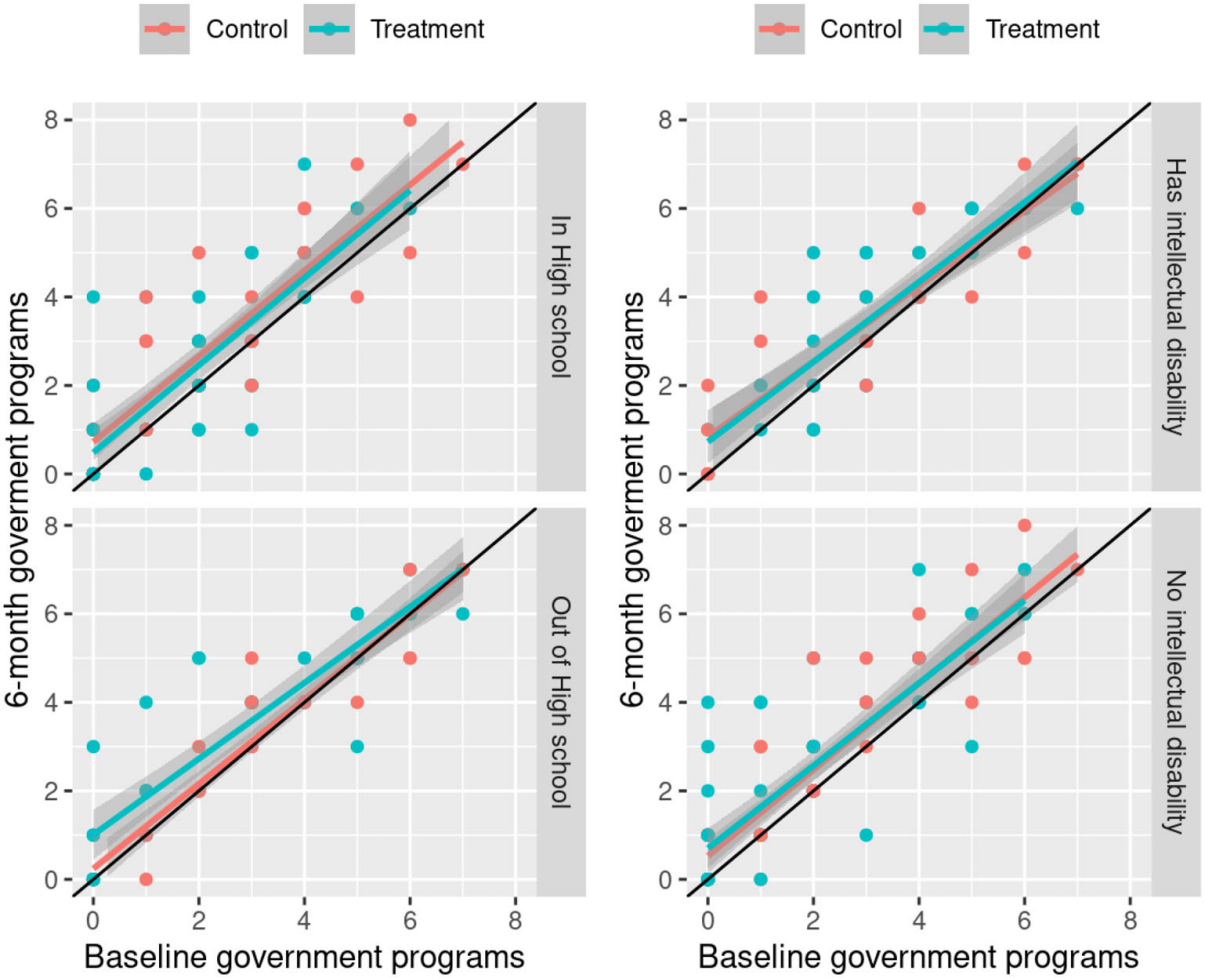
Scatter plots of baseline versus six‐month government programs, with estimated regression line by treatment group

To better understand changes in government programs from baseline to the six‐month follow‐up, Figure 2 depicts scatterplots with government programs received at baseline on the *x*‐axis and government programs received at the six‐month follow‐up on the y‐axis for each subgroup. The black line through the charts depicts the 1:1 line, meaning that youth were receiving the same number of government programs at baseline and at the six‐month follow‐up. Visual inspection of Figure 2 suggested that both treatment and control participants who were in high school at baseline (top left) were receiving more services at the six‐month follow‐up relative to baseline (i.e. the lines for both groups were above the black line). The significant treatment effect for those out of high school is depicted in the bottom left quadrant: Treatment effects on government programs were most pronounced for families who entered the study receiving fewer (versus more) programs. For both those who did and did not have intellectual disability (right half of the chart), the treatment and control estimated lines were above the black line, suggesting that both groups were receiving more government programs at the six‐month follow‐up than at baseline. For those with intellectual disability, this was most pronounced for youth receiving fewer government programs at baseline.

Table 4 presents estimates from the four regression models predicting government programs at the 12‐month follow‐up. As can be seen from the table, there was not a significant treatment effect on government programs at this timepoint for youth in high school at baseline, youth out of high school at baseline, or for youth with or without intellectual disability. Scatterplots with government programs received at baseline on the *x*‐axis, and government programs received at the 12‐month follow‐up on the *y*‐axis are presented in Supporting Information.

**Table 4:** Models examining treatment effects on government programs at 12‐month follow‐up, stratified by high school exit and intellectual disability status

(a) Stratified by high school exit						
	In high school			Out of high school		
	Odds Ratio	95% CI odds ratio	Chi‐square (*df*)	Odds ratio	95% CI odds ratio	Chi‐square (*df*)
Baseline government programs	5.52	3.70–8.22	70.38 (1)***	5.30	3.28–8.55	46.71 (1)***
Treatment vs. Control^r^ group	1.13	0.54–2.39	0.10 (1)	1.37	0.51–3.73	0.39 (1)
Site			2.67 (2)			0.99 (2)
Illinois vs. Tennessee^r^	1.73	0.70–4.28		0.95	0.28–3.24	
Wisconsin vs. Tennessee^r^	2.22	0.81–6.06		0.55	0.15–2.03	
Cohort one vs. Cohort two^r^	1.42	0.64–3.14	0.75 (1)	2.31	0.80–6.65	2.39 (1)

(b) Stratified by intellectual disability						
	Has intellectual disability			No intellectual disability		
	Odds ratio	95% CI odds ratio	Chi‐square (*df*)	Odds ratio	95% CI odds ratio	Chi‐square (*df*)
Baseline government programs	4.45	2.81–7.06	40.22 (1)***	5.06	3.45–7.41	69.35 (1)***
Treatment vs. Control^r^ group	1.38	0.53–3.60	0.44 (1)	0.99	0.46–2.15	0.01 (1)
Site			4.11 (2)			0.57 (2)
Illinois vs. Tennessee^r^	3.84	1.03–14.37		1.19	0.48–2.97	
Wisconsin vs. Tennessee^r^	1.47	0.46–4.70		1.49	0.53–4.19	
Cohort one vs. Cohort two^r^	1.04	0.39–2.78	0.01 (1)	1.88	0.86–4.11	2.47 (1)

CI, confidence interval; ^r^ = reference group.

*p < .05; **p < .01; ***, *p* < .001.

#### Direct services

Tables 5 and 6 present estimates from the four subgroup regression models predicting direct services at the six‐month follow‐up (Table 5) and the 12‐month follow‐up (Table 6). After controlling for baseline services, there was not a significant treatment group effect on direct services at six months or 12 months for any of the subgroups. Scatterplots with direct services received at baseline on the *x*‐axis and direct services received at the six‐month and 12‐month follow‐ups on the *y*‐axis are presented in Supporting Information.

**Table 5:** Models examining treatment effects on direct services at six‐month follow‐up, stratified by high school exit and intellectual disability status

(a) Stratified by high school exit						
	In high school			Out of high school		
	Odds ratio	95% CI odds ratio	Chi‐square (*df*)	Odds ratio	95% CI odds ratio	Chi‐square (*df*)
Baseline direct services	3.32	2.37–4.65	49.01 (1)***	3.69	2.48–5.48	41.39 (1)***
Treatment vs. Control^r^ group	1.43	0.69–2.95	0.93 (1)	1.67	0.69–4.04	1.27 (1)
Site			1.27 (2)			2.88 (2)
Illinois vs. Tennessee^r^	1.02	0.38–2.77		0.81	0.26–2.60	
Wisconsin vs. Tennessee^r^	1.63	0.60–4.39		2.03	0.69–5.97	
Cohort one vs. Cohort two^r^	0.53	0.25–1.13	2.66 (1)	1.31	0.51–3.39	0.32 (1)

(b) Stratified by intellectual disability						
	Has intellectual disability			No intellectual disability		
	Odds ratio	95% CI odds ratio	Chi‐square (*df*)	Odds ratio	95% CI odds ratio	Chi‐square (*df*)
Baseline direct services	3.06	2.04–4.59	29.11 (1)***	3.96	2.80–5.60	60.78 (1)***
Treatment vs. Control^r^ group	1.75	0.71–4.31	1.46 (1)	1.35	0.66–2.74	0.67 (1)
Site			0.37 (1)			6.53 (2)*
Illinois vs. Tennessee^r^	1.36	0.39–4.68		0.72	0.26–1.98	
Wisconsin vs. Tennessee^r^	1.37	0.45–4.11		2.24	0.89–5.63	
Cohort one vs. Cohort two^r^	0.86	0.32–2.32	0.08 (1)	0.56	0.27–1.14	2.57 (1)

CI, confidence interval; ^r^, reference group.

*p < .05; **p < .01; ***p < .001.

**Table 6:** Models examining treatment effects on direct services at 12‐month follow‐up, stratified by high school exit and intellectual disability status

(a) Stratified by high school exit						
	In high school			Out of high school		
	Odds ratio	95% CI odds ratio	Chi‐square (*df*)	Odds ratio	95% CI odds ratio	Chi‐square (*df*)
Baseline direct services	3.09	2.24–4.27	46.78 (1)***	3.09	2.12–4.53	33.89 (1)***
Treatment vs. Control^r^ group	1.11	0.54–2.28	0.08 (1)	0.57	0.23–1.43	1.42 (1)
Site			4.38 (2)			2.58 (2)
Illinois vs. Tennessee^r^	1.50	0.56–4.02		1.75	0.52–5.90	
Wisconsin vs. Tennessee^r^	2.89	1.05–7.99		2.79	0.80–9.78	
Cohort one vs. Cohort two^r^	0.62	0.29–1.30	1.64 (1)	0.63	0.24–1.65	0.89 (1)

(b) Stratified by intellectual disability						
	Has intellectual disability			No intellectual disability		
	Odds ratio	95% CI odds ratio	Chi‐square (*df*)	Odds ratio	95% CI odds ratio	Chi‐square (*df*)
Baseline direct services	2.65	1.79–3.93	23.66 (1)***	3.75	2.67–5.28	57.83 (1)***
Treatment vs. Control^r^ group	1.13	0.46–2.82	0.07 (1)	0.75	0.36–1.55	0.61 (1)
Site			0.44 (2)			8.61 (2)*
Illinois vs. Tennessee^r^	1.29	0.37–4.59		1.34	0.50–3.62	
Wisconsin vs. Tennessee^r^	1.46	0.47–4.54		3.94	1.42–10.95	
Cohort one vs. Cohort two^r^	0.81	0.31–2.10	0.19 (1)	0.46	0.22–0.95	4.36 (1)*

CI, confidence interval; ^r^, reference group.

*p < .05**; *p* < .01; ***p < .001.

### Other analyses

Other analyses, presented in S1, explore associations between treatment dose and service outcomes and present unadjusted group means and standard deviations for the service variables.

## Discussion

This study tested whether a parent advocacy intervention was effective in increasing service access for autistic youth who are transitioning to adulthood. Overall, we could not conclude that participation in ASSIST increased access to services. Secondary analyses, however, provided a more nuanced understanding of subgroups that may benefit from ASSIST. Specifically, there was some evidence for greater gains in government programs for families who took ASSIST (relative to the control condition) after their youth had exited high school. The increase in government programs, specifically, for those out of high school makes good sense given eligibility criteria for these programs. Though direct services (such as speech language services or educational services) may be easier to obtain before autistic youth leave high school (Shattuck et al., 2011), those in high school would not yet be eligible for some of the government programs aimed at adults with disabilities. It is generally acknowledged that transition planning should begin as early as possible to prepare youth for life after high school (e.g. Halpern, 1994; Suk, Martin, McConnell, & Biles, 2020). However, it remains unclear when it is most helpful to provide information on adult services – especially government programs for which youth may not yet be eligible. To understand how a program on adult services can benefit those in high school, it may be necessary to follow families for a longer period of time to see if and how they translate this earlier information into service access once their youth is eligible for more adult services. Interestingly, an examination of the scatterplots suggested that many families of youth in high school reported increases in government programs from baseline to follow‐up, but those increases did not differ by treatment group. It may be that written information on government programs (and not the full ASSIST class) is sufficient to support families of youth in high school in accessing the subset of government programs that they might be eligible for prior to high school exit.

One possible hypothesis for the lack of difference between treatment and control groups in the primary analyses is that the “active ingredient” of the intervention was comprehensive information about adult services, which was given to both groups. Specifically, we tested whether information plus explanation/discussion (i.e. the ASSIST course) was more effective in increasing service access relative to comprehensive written information on services (without explanation). Supporting analyses (Table S1, see also scatterplots in Figure 2) revealed that both the treatment and control groups were receiving more government programs at the follow‐up data collections than they were receiving at baseline. Though conclusions must be drawn cautiously without an untreated control group, these patterns suggest that some parents may have been able to leverage the information from comprehensive written materials to improve service access. It will be important, in future research, to understand characteristics of families who might be able to benefit from less intensive interventions such as sharing written resources, versus those who may need a 12‐week class to see increases in service access. It will also be important to test the effects of ASSIST against a control group that does not receive information, to determine if access to comprehensive information (regardless of format) is the mechanism through which ASSIST might improve service access.

Interpretation of study findings should consider the pervasive period effects that impacted intervention delivery and service access outcomes, specifically, the COVID‐19 pandemic. Multiple studies, including our own, found that families of individuals with disabilities struggled to access services during the pandemic (Burke et al., 2022; Goyal, Hunt, Kuper, Shakespeare, & Banks, 2023; Jeste et al., 2020). Government offices were closed for substantial periods of time, and processes to apply for services and programs – already confusing before the pandemic – became even more difficult to navigate as government agencies developed new processes for online applications and determining eligibility. Follow‐up data for the current project were collected during this change. It is unclear whether the effectiveness of ASSIST would be different if outcome data were collected during more typical times.

The period effects may also help to explain the disparate pattern of findings between government programs that fund services and direct services themselves. While it was rare for families to receive fewer government programs at follow‐up data collections relative to baseline (see Figure 2 and Supporting Information), it was more common for families to receive fewer direct services over time ‐ especially those who were receiving a higher number of services at baseline (see Supporting Information). This pattern is likely an effect of COVID‐19. Even when families were able to procure funding for services through new government programs, workforce shortages and social distancing requirements made it challenging to utilize that funding for services (Hewitt et al., 2020; Jeste et al., 2020). While we suspect this pattern of findings is pandemic‐specific, it may reflect broader challenges in translating funding into direct services. Future research is needed to understand the process from getting funding for services to receiving the services, as well as where breakdowns may happen in the process.

It may also be important to consider the area of the United States where a person lives when investigating the processes through which families translate government programs into direct services. Though the study site was a control variable in our models, we found that autistic youth in Wisconsin received more direct services than those in Tennessee, but not more government programs. This finding suggests that taking the statewide context into account will likely be important to understanding processes of translating government programs into direct services.

There are limitations to this study. First, intervention outcome data were collected during some of the most acute months of the COVID‐19 pandemic, when many in‐person activities (and the direct services that supported them) were paused. Thus, it is impossible to know the effectiveness of ASSIST during typical times. Second, our indicator of service access was parent report; we were unable to corroborate whether the services were received (e.g. though administrative databases). Because parents were not blinded to group assignment, those in the treatment group (vs. controls) may have been primed to identify the services that they received for outcome reporting. This could mean that positive results reflect detection bias and not a treatment effect. Further, counts of services received (i.e. the outcome in this study) assume that all services are equal and do not account for the extent to which each service is experienced as beneficial. A full consideration of the complexities of measuring service access can be found in Burke, Cheung, Best, DaWalt, and Taylor (2024). Third, the racial/ethnic and socioeconomic representativeness of the sample was limited. It is unclear whether ASSIST would be more or less effective among families of autistic youth who tend to be the most underserved, such as those who identify as Black or Latino (Magaña, Lopez, Aguinaga, & Morton, 2013; Roux et al., 2024). Finally, the intervention and service outcomes are specific to the United States context and likely do not generalize to other national contexts.

These limitations are offset by important strengths. The efficacy of ASSIST was tested using a rigorous RCT design. Participants were recruited from three states in the United States, which increases the likelihood that findings are not limited to one state's adult service system. Finally, the inclusion of both government programs and direct services is a novel approach to measuring services (nearly all prior studies choose one or the other) and allowed us to uncover information about how procuring government programs that fund services may not necessarily lead to a greater number of services received.

## Conclusion

ASSIST may be effective in improving access to state and federal programs that fund services for families of autistic youth who are out of high school; however, there was no evidence for intervention effects on the direct services themselves. Future research is needed to understand and mitigate the barriers to leveraging parent advocacy into improved service access, as well as the barriers to translating funding from government programs into better access to the direct services funded by those programs.

## Ethics statement

Written consent was obtained from all parents, and consent (if the youth was over the age of 18 and their own legal guardian) or assent was obtained from youth participants. The Institutional Review Boards at the participating study sites approved all study procedures (single IRB; Vanderbilt University Medical Center IRB of record approval number 191187 granted on October 4, 2019).

## Trial registration

The trial was registered in ClinicalTrials.gov, # NCT04173663, and was first posted on the website November 22, 2019. Registration information can be found at https://clinicaltrials.gov/study/NCT04173663.

**Key points**

**What's known**

- Access to adult disability services can support work, postsecondary education, and community integration for autistic youth. However, adult disability services are difficult to access and complicated to understand, leaving many youth underserved.

**What's new**

- ASSIST is a parent advocacy intervention that teaches parents of autistic youth how to access adult services and supports. For families of autistic youth who have left high school, taking ASSIST may increase access to government programs that fund services but not to the direct services themselves.

**What's relevant**

- Though there was some evidence that ASSIST might facilitate access to services for some youth, a different approach may be necessary to more robustly connect youth to services.

## Supporting information


**Table S1.** Unadjusted means and standard deviations, paired t‐values, and effect sizes for change in services within treatment groups.
**Table S2**. Number of autistic youth out of high school who received each government program at baseline and at the six‐month follow‐up.
**Table S3**. Partial correlations between the number of ASSIST sessions attended and services for the full treatment group and for subsamples, controlling for baseline services.
**Table S4**. Unadjusted means and standard deviations by treatment group for services at each time point for each subsample.
**Figure S1**. Scatter plots of baseline versus 12‐month government programs for subgroups, with estimated regression line by treatment group.
**Figure S2**. Scatter plots of baseline versus six‐month direct services for subgroups, with estimated regression line by treatment group.
**Figure S3**. Scatter plots of baseline versus 12‐month direct services for subgroups, with estimated regression line by treatment group.

## Data Availability

All data, including raw and summary variables and variable guides, are publicly available in the National Institute of Mental Health Data Archive.

## Endnote

1 We had initially planned to do in‐person diagnostic confirmation of autism for all participants but were required to adjust our study design once social distancing regulations were put into place due to COVID‐19. The Social Communication Questionnaire and evidence of a previous autism diagnosis were initially used to screen out those who were unlikely to meet criteria for autism in a lab‐administered diagnostic evaluation and thus would unnecessarily come for the in‐person visit. When we were no longer able to do in‐person visits, we continued to use the Social Community Questionnaire and documentation of a previous autism diagnosis as a screener to indicate that the youth would likely meet diagnostic criteria for autism.

## References

[jcpp70036-bib-0001] Alverson, C.Y. , & Yamamoto, S.H. (2016). Employment outcomes of vocational rehabilitation clients with autism Spectrum disorders. Career Development and Transition for Exceptional Individuals, 40, 144–155.

[jcpp70036-bib-0002] Burke, M. , Cheung, W.C. , Best, M. , DaWalt, L.S. , & Taylor, J.L. (2024). Measuring what matters: Considerations for the measurement of services for individuals with autism. Journal of Developmental and Physical Disabilities, 36, 423–439.

[jcpp70036-bib-0003] Burke, M.M. , Cheung, W. , Li, C. , DaWalt, L. , Segal, J. , & Taylor, J.L. (2022). Parental perceptions of service access for transition‐aged youth with autism during COVID‐19. Intellectual and Developmental Disabilities, 60, 369–381. 36162047 10.1352/1934-9556-60.5.369 PMC9942276

[jcpp70036-bib-0004] Burke, M.M. , Goldman, S.E. , & Li, C. (2023). A tale of two adaptations of a special education advocacy program. Intellectual and Developmental Disabilities, 61, 95–109. 36996282 10.1352/1934-9556-61.2.95

[jcpp70036-bib-0005] Gilson, C.B. , Bethune, L.K. , Carter, E.W. , & McMillan, E.D. (2017). Informing and equipping parents of people with intellectual and developmental disabilities. Intellectual and Developmental Disabilities, 55, 347–360. 28972871 10.1352/1934-9556-55.5.347

[jcpp70036-bib-0006] Goldman, S.E. , Goscicki, B.L. , Burke, M.M. , & Hodapp, R.M. (2020). Developing special education advocates: What changes during an advocacy training program? Journal of Policy and Practice in Intellectual Disabilities, 17, 308–317.

[jcpp70036-bib-0007] Goyal, D. , Hunt, X. , Kuper, H. , Shakespeare, T. , & Banks, L.M. (2023). Impact of the COVID‐19 pandemic on people with disabilities and implications for health services research. Journal of Health Services Research & Policy, 28, 77–79. 36821779 10.1177/13558196231160047 PMC9968687

[jcpp70036-bib-0008] Halpern, A.S. (1994). The transition of youth with disabilities to adult life: A position statement of the division on career development and transition, the Council for Exceptional Children. Career Development for Exceptional Individuals, 17, 115–124.

[jcpp70036-bib-0009] Hewitt, A. , Pettingell, S. , Kramme, J. , Smith, J.D. , Dean, K. , & Kleist, B. (2020). The Direct Support Workforce and COVID‐19 National Survey Report 2020. Institute on Community Integration, University of Minnesota. Available from: http://idea.ed.gov/uploads/finalregulations.pdf

[jcpp70036-bib-0010] Hollocks, M.J. , Lerh, J.W. , Magiati, I. , Meiser‐Stedman, R. , & Brugha, T.S. (2019). Anxiety and depression in adults with autism spectrum disorder: A systematic review and meta‐analysis. Psychological Medicine, 49, 559–572. 30178724 10.1017/S0033291718002283

[jcpp70036-bib-0011] IOM (Institute of Medicine) , & NRC (National Research Council) . (2014). Investing in the health and well‐being of young adults. Washington, DC: The National Academies Press.

[jcpp70036-bib-0012] Jeste, S. , Hyde, C. , Distefano, C. , Halladay, A. , Ray, S. , Porath, M. , … & Thurm, A. (2020). Changes in access to educational and healthcare services for individuals with intellectual and developmental disabilities during COVID‐19 restrictions. Journal of Intellectual Disability Research, 64, 825–833. 32939917 10.1111/jir.12776

[jcpp70036-bib-0013] Lai, J.K.Y. , & Weiss, J.A. (2017). Priority service needs and receipt across the lifespan for individuals with autism spectrum disorder. Autism Research, 10, 1436–1447. 28383156 10.1002/aur.1786 PMC5573942

[jcpp70036-bib-0014] Laxman, D.J. , Taylor, J.L. , DaWalt, L.S. , Greenberg, J.S. , & Mailick, M.R. (2019). Loss in services precedes high school exit for teens with autism spectrum disorder: A longitudinal study. Autism Research, 12, 911–921. 31033222 10.1002/aur.2113 PMC6546537

[jcpp70036-bib-0015] Lee, C.E. , Burke, M.M. , DaWalt, L.S. , Li, C. , & Taylor, J.L. (2022). The role of parental advocacy in addressing service disparities for transition‐aged youth on the autism spectrum. Autism, 26, 1001–1006. 34841922 10.1177/13623613211057660 PMC9010347

[jcpp70036-bib-0016] Magaña, S. , Lopez, K. , Aguinaga, A. , & Morton, H. (2013). Access to diagnosis and treatment services among latino children with autism spectrum disorders. Intellectual and Developmental Disabilities, 51, 141–153. 23834211 10.1352/1934-9556-51.3.141

[jcpp70036-bib-0017] Newman, L. , Wagner, M. , Knokey, A.‐M. , Marder, C. , Nagle, K. , Shaver, D. , … & National Center for Special Education Research . (2011). The post‐high school outcomes of young adults with disabilities up to 8 years after high school. A report from the National Longitudinal Transition Study‐2. NCSER 2011‐3005.

[jcpp70036-bib-0018] Orsmond, G.I. , Shattuck, P.T. , Cooper, B.P. , Sterzing, P.R. , & Anderson, K.A. (2013). Social participation among young adults with an autism Spectrum disorder. Journal of Autism and Developmental Disorders, 43, 2710–2719. 23615687 10.1007/s10803-013-1833-8 PMC3795788

[jcpp70036-bib-0019] Roux, A.M. , Rast, J.E. , Anderson, K.A. , Garfield, T. , & Shattuck, P.T. (2021). Vocational rehabilitation service utilization and employment outcomes among secondary students on the autism Spectrum. Journal of Autism and Developmental Disorders, 51, 212–226. 32399821 10.1007/s10803-020-04533-0

[jcpp70036-bib-0020] Roux, A.M. , Voltaire, S. , Steinberg, H. , Williams, E.D. , Anderson, K.A. , Hutson, T.M. , & Shea, L.L. (2024). More than just a variable: The need to explicitly focus on black youth within autism transitions research. Autism in Adulthood, 6, 119–127. 39144071 10.1089/aut.2023.0041 PMC11320561

[jcpp70036-bib-0021] Rutter, M. , Bailey, A. , & Lord, C. (2003). SCQ: Social Communication Questionnaire. Los Angeles: Western Psychological Services.

[jcpp70036-bib-0022] Shattuck, P.T. , Narendorf, S.C. , Cooper, B. , Sterzing, P.R. , Wagner, M. , & Taylor, J.L. (2012). Postsecondary education and employment among youth with an autism spectrum disorder. Pediatrics, 129, 1042–1049. 22585766 10.1542/peds.2011-2864 PMC3362908

[jcpp70036-bib-0023] Shattuck, P.T. , Wagner, M. , Narendorf, S. , Sterzing, P. , & Hensley, M. (2011). Post‐high school service use among young adults with an autism spectrum disorder. Archives of Pediatrics & Adolescent Medicine, 165, 141–146. 21300654 10.1001/archpediatrics.2010.279 PMC3097532

[jcpp70036-bib-0024] Sparrow, S.S. , Saulnier, C.A. , Cicchetti, D.V. , & Doll, E.A. (2016). Vineland‐3: Vineland adaptive behavior scales. Manual. Pearson Assessments.

[jcpp70036-bib-0025] Suk, A.L. , Martin, J.E. , McConnell, A.E. , & Biles, T.L. (2020). States decrease their required secondary transition planning age: Federal Policy Must Change. Journal of Disability Policy Studies, 31, 112–118.

[jcpp70036-bib-0026] Szlamka, Z. , Tekola, B. , Hoekstra, R. , & Hanlon, C. (2022). The role of advocacy and empowerment in shaping service development for families raising children with developmental disabilities. Health Expectations, 25, 1882–1891. 35644908 10.1111/hex.13539 PMC9327816

[jcpp70036-bib-0027] Taylor, J.L. , DaWalt, L.S. , Burke, M.M. , Slaughter, J.C. , & Xu, M. (2023). Improving parents' ability to advocate for services for youth with autism: A randomized clinical trial. Autism Research, 16, 1976–1988. 37551665 10.1002/aur.3001 PMC10615697

[jcpp70036-bib-0028] Taylor, J.L. , & Henninger, N.A. (2015). Frequency and correlates of service access among youth with autism transitioning to adulthood. Journal of Autism and Developmental Disorders, 45, 179–191. 25081594 10.1007/s10803-014-2203-x PMC4288981

[jcpp70036-bib-0029] Taylor, J.L. , Hodapp, R.M. , Burke, M.M. , Waitz‐Kudla, S.N. , & Rabideau, C. (2017). Effects of a parent‐training intervention on service access and employment for youth with ASD. San Francisco, CA: International Meeting for Autism Research.

[jcpp70036-bib-0030] Taylor, J.L. , & Mailick, M.R. (2014). A longitudinal examination of 10‐year change in vocational and educational activities for adults with autism spectrum disorders. Developmental Psychology, 50, 699–708. 24001150 10.1037/a0034297 PMC3951267

[jcpp70036-bib-0031] Taylor, J.L. , Pezzimenti, F. , Burke, M.M. , DaWalt, L.S. , Lee, C.e. , & Rabideau, C. (2022). Development, feasibility, and acceptability of a nationally relevant parent training to improve service access during the transition to adulthood for youth with ASD. Journal of Autism and Developmental Disorders, 52, 2388–2399. 34232419 10.1007/s10803-021-05128-z PMC8262127

[jcpp70036-bib-0032] Taylor, J.L. , & Seltzer, M.M. (2012). Developing a vocational index for adults with autism spectrum disorders. Journal of Autism and Developmental Disorders, 42, 2669–2679. 22466690 10.1007/s10803-012-1524-x PMC3484183

[jcpp70036-bib-0033] Taylor, S. , Wright, A.C. , Pothier, H. , Hill, C. , & Roserberg, M. (2019). It's like I have an advantage in all this: Experiences of advocacy by parents of children with disabilities from professional backgrounds. Journal of Sociology and Social Welfare, 46, 159–183.

[jcpp70036-bib-0034] Trainor, A.A. (2008). Diverse approaches to parent advocacy during special education home—School interactions: Identification and use of cultural and social capital. Remedial and Special Education, 31, 34–47.

[jcpp70036-bib-0035] Turnbull, A.P. , & Turnbull, H.R. (2001). Families, professionals, and exceptionality: Collaborating for empowerment (4th edn). Upper Saddle River, NJ: Merrill Prentice Hall.

[jcpp70036-bib-0036] Wechsler, D. (1999). Wechsler abbreviated scales of intelligence (WASI) manual. San Antonio, TX: The Psychological Corporation.

