# Supplementary material for: Effects of a parent advocacy intervention on service access for transition‐aged autistic youth: a multisite randomized controlled trial
Source: J Child Psychol Psychiatry. 2025 Aug 17;67(2):212–24. doi: 10.1111/jcpp.70036 (PMC12582376; doi:10.1111/jcpp.70036)
Supplement: Supplementary file 1 — Table S1. Unadjusted means and standard deviations, paired t‐values, and effect sizes for change in services within treatment groups. Table S2. Number of autistic youth out of high school who received each government program at baseline and at the six‐month follow‐up. Table S3. Partial correlations between the number of ASSIST sessions attended and services for the full treatment group and for subsamples, controlling for baseline services. Table S4. Unadjusted means and standard deviations by treatment group for services at each time point for each subsample. Figure S1. Scatter plots of baseline versus 12‐month government programs for subgroups, with estimated regression line by treatment group. Figure S2. Scatter plots of baseline versus six‐month direct services for subgroups, with estimated regression line by treatment group. Figure S3. Scatter plots of baseline versus 12‐month direct services for subgroups, with estimated regression line by treatment group. [file JCPP-67-212-s001.docx]

**SUPPORTING INFORMATION**

**Change over time in treatment and control groups**

Our primary analyses provided strong tests of whether there were statistically significant differences in services between treatment and control groups at the six- and 12-month follow-ups, but they are less informative to understanding how each of these groups changed over time. For example, failing to find a statistically significant difference between groups in services at six-months could be because *neither* the treatment nor control groups were receiving more services, or it could be because *both* the treatment and control groups were receiving more services. To better understand change over time in services, we conducted follow-up analyses using paired sample t-tests within each group (see Table S1). Both the treatment group and control group had significant gains in the number of government programs from baseline to the six-month follow-up and to the 12-month follow-up, with medium effect sizes. In terms of direct services, there was primarily a pattern of no significant changes over time, though we did detect a decline in direct services for the treatment group (only) from baseline to the 12-month follow-up.

**Frequencies of gains in individual government programs at six-month follow-up for families of youth who have exited high school**

To further understand the treatment effect on government services at the six-month follow-up for families of youth who had exited high school, we examined the number of youth who were receiving each government program at baseline and six-month follow-up, stratified by treatment versus control group. Frequencies of each service for youth out of high school are presented in Table S2. As can be seen from the table, the overall increase in government programs in the treatment group relative to the control group was accounted for by a variety of government programs (as opposed to one particular program). Overall, the gains seemed to be mostly accounted for by increases in income supports [Supplemental Security Income (SSI), Social Security Disability Insurance (SSDI)] and public health insurance. That is, six of 35 youth (or 17%) from the intervention group were newly receiving SSI or SSDI, whereas one youth in the control group was newly receiving SSDI and one was no longer receiving SSI (for a net gain of 0 between these two types of income supports). Four youth in the treatment group (11.4%) were newly receiving government health insurance (Medicaid and/or Medicare) versus one youth in the control group (3.1%). Other smaller, but relative gains for the treatment group were noted in Vocational Rehabilitation services, legal protections, Medicaid waiver services, and Supplemental Nutrition Assistance Programs (SNAP). No families were receiving housing vouchers at either time point.

**Distribution of baseline versus 12-month government programs**

Scatterplots with government programs received at baseline on the x-axis, and government programs received at the 12-month follow-up on the y-axis for subgroups (i.e., those in high school, those who have exited of high school, those with intellectual disability, those without intellectual disability) are depicted in Figure S1. Similar to what was observed with the six-month government program data, visual inspection of the data suggested that families of youth in high school were receiving more government programs at the 12-month follow-up regardless of intervention group. Across all subgroups, those receiving fewer government programs at baseline appeared to have greater gains over the following year (regardless of treatment), compared to those receiving more government programs at baseline.

**Distribution of baseline versus follow-up direct services**

Scatterplots with direct services received at baseline on the x-axis plotted against direct services received at the six-month follow-up (Figure S2) or 12-month follow-up (Figure S3) on the y-axis were examined. Whereas the plotted regression lines were generally above the 1:1 line for government programs (indicating increases in government programs at follow-up relative to baseline), visual inspection suggested different patterns of change for direct services. For both the six-month and 12-month follow-ups, across all subgroups, there were generally increases in the number of services received over time for both treatment and control groups among those who started out with low numbers of services. However, for those who started out with a higher number of services, we observed that many – regardless of treatment group – were receiving fewer services at the six-month and the 12-month follow-ups.

**Exploring associations between treatment dose and service outcomes**

The analyses used to test the research questions of interest were intent-to-treat, with all participants included regardless of the number of ASSIST sessions that the treatment group attended (session attendance was reported in Taylor et al., 2022, which focused on implementation outcomes). Though full consideration of dosage effects is beyond the scope of this report, to inform future work we explored potential bivariate associations between the number of sessions that treatment group participants attended and the number of services at the six-month and 12-month follow-ups, controlling for the number of services at baseline. Partial correlations are reported in Table S3. As can be seen from the table, there were no significant associations between attendance and follow-up services after controlling for baseline services. This may be due, at least in part, to limited variability in attendance; 85% of participants attended at least three-quarters of the sessions (Taylor et al., 2022). Future analyses will delve more deeply into attendance to understand if there are certain sessions in which attendance is linked to gains in the specific services that were covered in that session.

**Unadjusted group means and standard deviations**

Unadjusted means and standard deviations for service variables by treatment group for each subsample are presented in Table S4 (means and standard deviations by treatment group for the full sample are presented in Table S1).

Table S1

Unadjusted means and standard deviations, paired t-values, and effect sizes for change in services within treatment groups

|  | Means (standard deviations) | | |  | Baseline to six-month change | |  | Baseline to 12-month change | |
| --- | --- | --- | --- | --- | --- | --- | --- | --- | --- |
|  | Baseline Services | Six-month Services | 12-month Services |  | Paired  t-value | Cohen’s d |  | Paired  t-value | Cohen’s d |
| Government Programs |  |  |  |  |  |  |  |  |  |
| Treatment | 2.41 (2.05) | 2.88 (2.17) | 3.03 (2.09) |  | 4.26*** | 0.47 |  | 4.19*** | 0.47 |
| Control | 2.59 (2.11) | 3.08 (2.16) | 2.91 (2.17) |  | 4.24*** | 0.48 |  | 3.33** | 0.38 |
| Direct Services |  |  |  |  |  |  |  |  |  |
| Treatment | 4.52 (2.64) | 4.40 (2.76) | 4.01 (2.93) |  | -0.35 | -0.04 |  | -2.93** | -0.33 |
| Control | 4.69 (3.03) | 4.33 (2.74) | 4.33 (2.98) |  | -1.38 | -0.15 |  | -1.59 | -0.18 |

*p < .05 ** p < .01 *** p < .001

Table S2

Number of autistic youth out of high school who received each government program at baseline and at the six-month follow-up

|  | Treatment (n = 35) | |  | Control (n = 32) | |
| --- | --- | --- | --- | --- | --- |
|  | N receiving at Baseline | N receiving at 6-month |  | N receiving at Baseline | N receiving at 6-month |
| Supplemental Security Income (SSI) | 16 | 18 |  | 18 | 17 |
| Social Security Disability Insurance (SSDI) | 5 | 9 |  | 8 | 9 |
| Vocational Rehabilitation (VR) services | 15 | 17 |  | 10 | 11 |
| Legal Protections (including conservatorship, guardianship, power of attorney, health care surrogate) | 14 | 17 |  | 21 | 22 |
| Special Needs Trust and ABLE accounts | 11 | 12 |  | 16 | 16 |
| Housing Choice Voucher (Section 8) | 0 | 0 |  | 0 | 0 |
| Supplemental Nutrition Assistance Program (SNAP) | 6 | 9 |  | 2 | 4 |
| Medicaid or Medicare Health Insurance | 21 | 25 |  | 21 | 22 |
| Medicaid Waiver Services or Medicaid Long Term Services and Support | 13 | 15 |  | 16 | 14 |

Figure S1. Scatter plots of baseline versus 12-month government programs for subgroups, with estimated regression line by treatment group


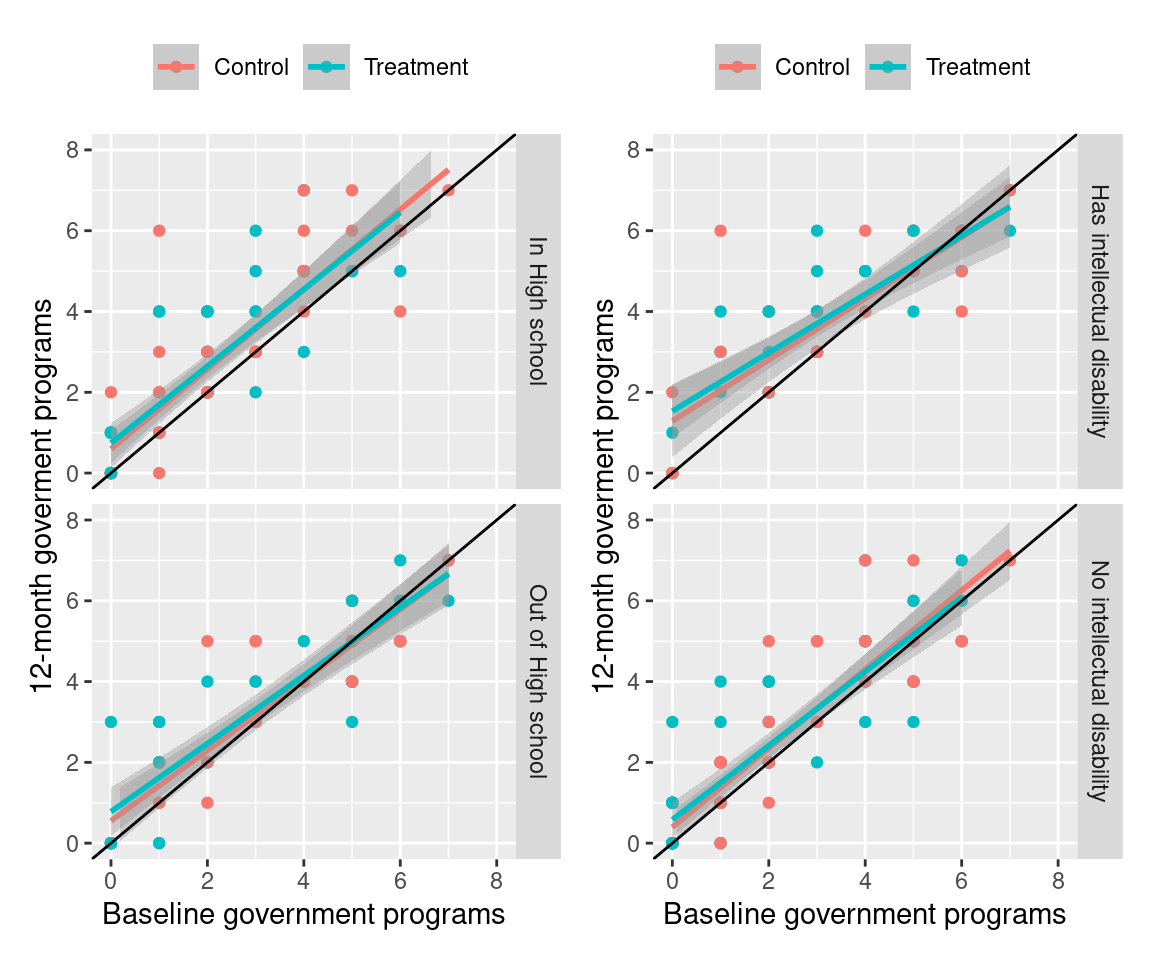


Figure S2. Scatter plots of baseline versus six-month direct services for subgroups, with estimated regression line by treatment group


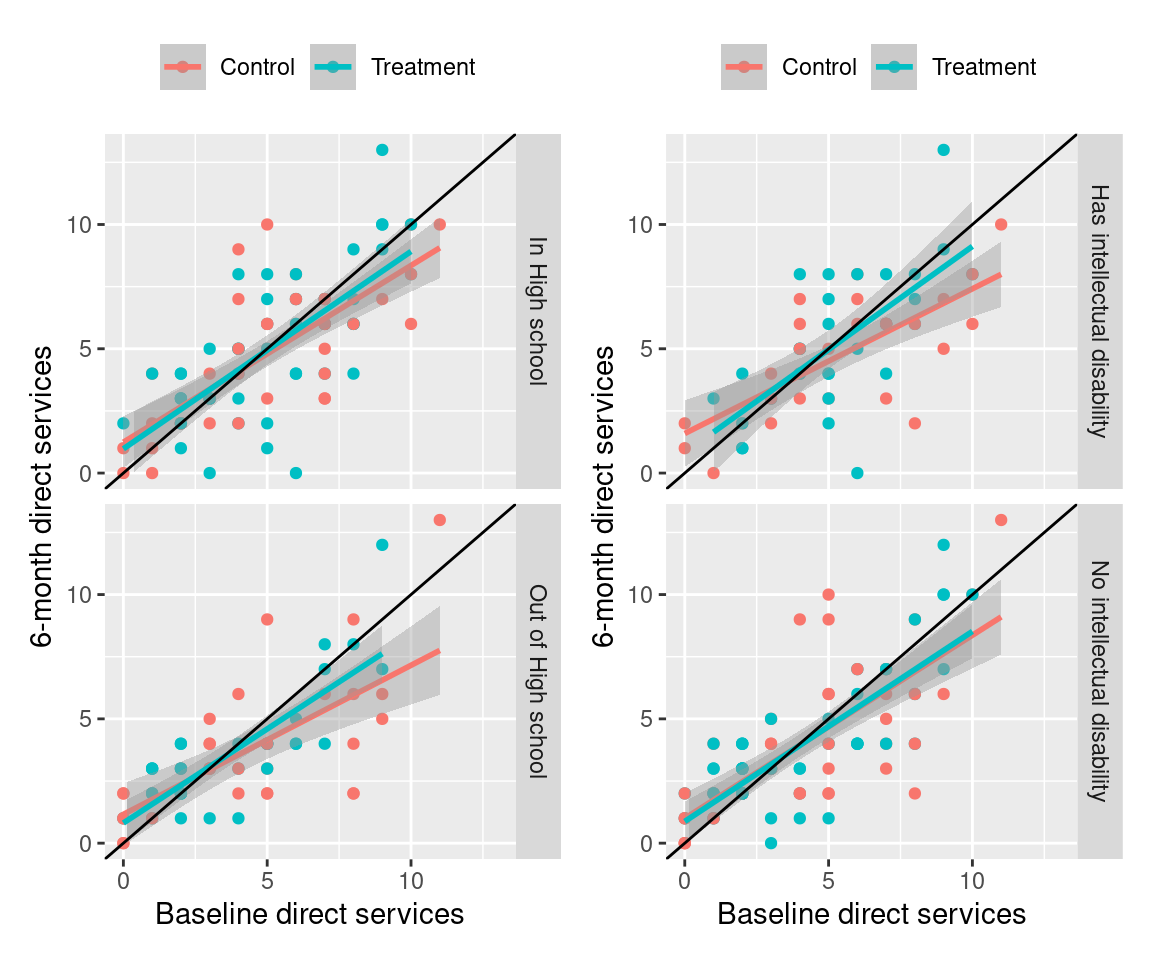


Figure S3. Scatter plots of baseline versus 12-month direct services for subgroups, with estimated regression line by treatment group


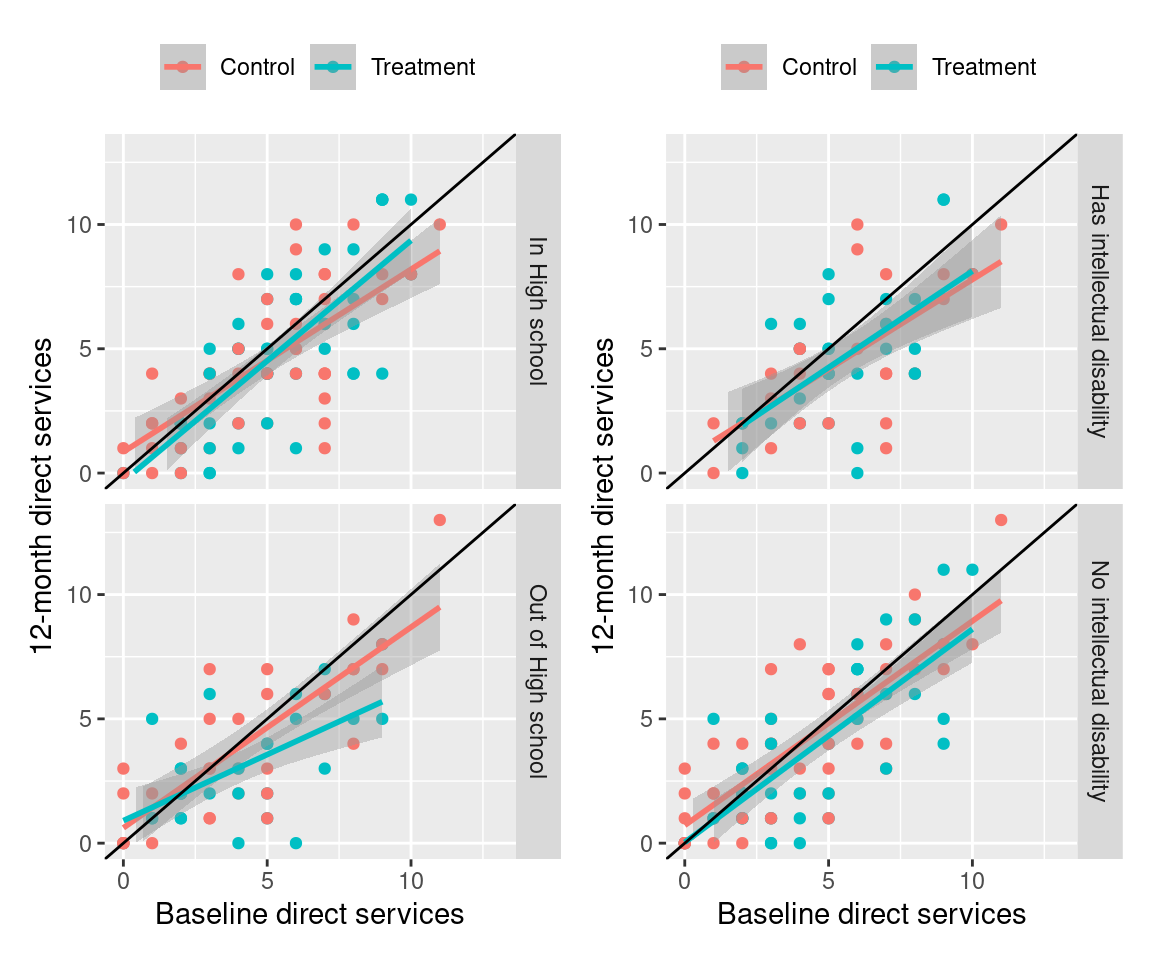


Table S3

Partial correlations between the number of ASSIST sessions attended and services for the full treatment group and for subsamples, controlling for baseline services

|  | Full group | Subsamples | | | |
| --- | --- | --- | --- | --- | --- |
|  |  | Those in high school | Those out of high school | Those with intellectual disability | Those without intellectual disability |
| Six-month government programs | 0.08 | 0.04 | 0.11 | 0.23 | -0.03 |
| 12-month government programs | 0.10 | 0.16 | -0.02 | 0.20 | -0.01 |
| Six-month direct services | 0.07 | 0.12 | -0.08 | 0.14 | -0.04 |
| 12-month direct services | 0.10 | 0.15 | -0.03 | 0.11 | 0.10 |

Note. The relevant baseline service variable was statistically controlled in all analyses (e.g., baseline government programs were controlled for the correlation between attendance and 6-month government programs). All correlations include only treatment group participants.

Table S4

Unadjusted means and standard deviations by treatment group for services at each time point for each subsample

|  |  | Baseline government programs  M (SD) | Six-month government programs  M (SD) | 12-month government programs  M (SD) | Baseline direct services  M (SD) | Six-month direct services  M (SD) | 12-month direct services  M (SD) |
| --- | --- | --- | --- | --- | --- | --- | --- |
| In high school | Treatment | 2.08 (1.69) | 2.44 (1.96) | 2.79 (1.92) | 5.10 (2.59) | 4.98 (2.90) | 4.66 (3.21) |
|  | Control | 2.08 (2.00) | 2.72 (2.18) | 2.61 (2.21) | 5.00 (2.84) | 4.77 (2.65) | 4.51 (2.88) |
|  |  |  |  |  |  |  |  |
| Out of high school | Treatment | 2.89 (2.43) | 3.49 (2.33) | 3.39 (2.30) | 3.69 (2.53) | 3.60 (2.37) | 3.03 (2.17) |
|  | Control | 3.35 (2.07) | 3.59 (2.06) | 3.40 (2.06) | 4.24 (3.29) | 3.69 (2.80) | 4.03 (3.16) |
|  |  |  |  |  |  |  |  |
| Has intellectual disability | Treatment | 3.24 (1.87) | 3.58 (1.95) | 3.91 (1.63) | 5.27 (2.31) | 5.13 (2.84) | 4.56 (2.76) |
|  | Control | 3.19 (2.10) | 3.53 (2.01) | 3.70 (1.96) | 5.53 (2.99) | 4.83 (2.34) | 4.74 (2.89) |
|  |  |  |  |  |  |  |  |
| Does not have intellectual disability | Treatment | 1.88 (2.01) | 2.46 (2.21) | 2.41 (2.17) | 4.04 (2.75) | 3.96 (2.64) | 3.63 (3.02) |
|  | Control | 2.23 (2.05) | 2.80 (2.23) | 2.50 (2.18) | 4.19 (2.96) | 4.02 (2.95) | 4.11 (3.03) |
